# Lung transplant outcomes for recipients with alpha-1 antitrypsin deficiency, by use of alpha-1 antitrypsin augmentation therapy

**DOI:** 10.1016/j.jhlto.2024.100201

**Published:** 2024-12-24

**Authors:** Atharv V. Oak, Jessica M. Ruck, Alfred J. Casillan, Armaan F. Akbar, Ramon A. Riojas, Pali D. Shah, Jinny S. Ha, Sara Strout, Allan B. Massie, Dorry L. Segev, Christian A. Merlo, Errol L. Bush

**Affiliations:** a Department of Surgery, Johns Hopkins University School of Medicine, Baltimore, Maryland; b Department of Medicine, Johns Hopkins University School of Medicine, Baltimore, Maryland; c Department of Pharmacy, Johns Hopkins University School of Medicine, Baltimore, Maryland; d Department of Surgery, New York University Grossman School of Medicine and Langone Health, New York, New York; e Department of Population Health, New York University Grossman School of Medicine and Langone Health, New York, New York; f Scientific Registry of Transplant Recipients, Minneapolis, Minnesota

**Keywords:** alpha-1 antitrypsin, emphysema, lung transplant, augmentation therapy, survival outcomes

## Abstract

**Background:**

For patients with alpha-1 antitrypsin (AAT) deficiency, AAT augmentation therapy can be an important part of care. However, for those who require a lung transplant (LT), there is currently only limited information to guide the use of AAT augmentation therapy post-LT.

**Methods:**

We identified all LT recipients from 2011-2021 in the Scientific Registry of Transplant Recipients with an AAT deficiency diagnosis. We categorized recipients by use of AAT augmentation therapy post-LT and compared their baseline characteristics using Fisher’s exact test and Wilcoxon rank-sum tests. We used Kaplan-Meier analyses and estimated the average treatment effect (ATE) of post-LT AAT augmentation therapy on mortality and all-cause graft failure (ACGF). The ATE measures the observed effect we would see if everyone in the population received the intervention as opposed to just a subset.

**Results:**

Among the 447 recipients with AAT deficiency, 109 used AAT augmentation therapy pre-LT, of which 32 (29.4%) continued post-LT. Recipients who used augmentation therapy post-LT were younger (56.5 [53-59.75] vs 57 [53.75-63], *p* = 0.04) and had shorter ischemia time (mean 311 vs 363 minutes, *p* = 0.03) than those who did not. The age-adjusted ATE estimate of post-LT augmentation therapy use on time to death and ACGF was +1.69 and +1.48 years, respectively. Post-LT augmentation therapy use was associated with a mortality reduction in the top quartile bilirubin subgroup (*p* = 0.02, log-rank test).

**Conclusions:**

In our study, the use of augmentation therapy post-LT was associated with improved survival. Confirmatory prospective studies should be considered to inform post-LT AAT therapy guidelines.

## Background

Alpha-1 antitrypsin (AAT) deficiency is one of the most common genetic respiratory disorders, with a prevalence of around 1 in 3,000 people in North America.^1^ AAT is a neutrophil elastase inhibitor synthesized primarily in the liver; deficiency of this antiprotease results in the degradation of type IV collagen and elastin. As a result, AAT deficiency commonly results in emphysema, liver dysfunction, necrotizing panniculitis, and other inflammatory and degenerative conditions.^2^ Treatment can include AAT augmentation therapy, in which a patient receives weekly infusions of AAT antiprotease from pooled plasma to achieve normal plasma levels and slow disease progression.^3^, ^4^ While AAT antiprotease augmentation therapy can slow lung damage and ameliorate serum abnormalities, some patients continue to progress to end-stage lung disease due to emphysematous changes and require lung transplantation (LT).^5^

While LT restores respiratory function, recipients with AAT deficiency (compared to chronic obstructive pulmonary disease as an indication) have been found to have increased risk of post-LT complications, including airway dehiscence and requiring treatment for rejection within 1 year of transplant.^6^ They also can continue to have AAT-related problems in other organs, particularly the liver.^6^, ^7^, ^8^ Some reports have suggested that post-LT outcomes might be improved by continuing AAT augmentation therapy post-LT.^7^ However, there have been no national studies of AAT augmentation therapy continuation to inform broader changes in therapy continuation recommendations.^7^ Current American Thoracic Society/European Respiratory Society guidelines do not have firm recommendations for use of antiprotease augmentation therapy post-LT due to the limited availability of rigorous clinical evidence.^9^

It is unknown whether AAT augmentation therapy improves mortality or graft maintenance post-LT. Therefore, we used national registry data to retrospectively evaluate outcomes of AAT-deficient recipients by AAT augmentation therapy use pre- and post-LT.

## Methods

### Data source

This study used data from the United States Scientific Registry of Transplant Recipients (SRTR). The SRTR data system includes data on all donors, wait-listed candidates, and transplant recipients in the United States, submitted by the members of the Organ Procurement and Transplantation Network. The Health Resources and Services Administration, US Department of Health and Human Services provides oversight to the activities of the Organ Procurement and Transplantation Network and SRTR contractors. These data have been described elsewhere.^10^ Variables available in the SRTR dataset can be viewed at https://www.srtr.org/requesting-srtr-data/saf-data-dictionary/.

Data regarding outpatient pharmacy prescriptions were obtained from a US pharmaceutical claims warehouse. Prescription fill data were deidentified before receipt with a unique patient identifier linking each prescription fill to the corresponding patient in the SRTR database. The pharmacy fill records were based on billing claims, including self-paid bills and those reimbursed by public and private payers. These records were aggregated from sources, including claims warehouses, retail pharmacies, and prescription benefit managers, and captured approximately 60% of US retail pharmacy transactions. Prescription information was available for approximately 74% of all LT recipients in SRTR and for approximately 78% of recipients with AAT deficiency.

### Study population

Using SRTR data, we identified all adult (>18 years old) LT recipients with a diagnosis of AAT deficiency (diagnosis code 1606: LU: ALPHA-1-ANTITRYPSIN DEFICIENCY) who were transplanted from January 2011 through December 2021 who also had outpatient prescription fill data available. We then categorized recipients by their use of AAT augmentation therapy based on the presence and timing of prescription fills for AAT augmentation therapy medications. Recipients were grouped as (1) no AAT augmentation therapy use (“no augmentation”), (2) augmentation therapy use both pre- and post-LT (“augmentation continuation”), (3) augmentation therapy use pre-LT only (“augmentation discontinuation”), and (4) augmentation therapy use post-LT only (“augmentation commencement post-LT”). Of note, to minimize misclassification, we excluded recipients who died during the transplant hospitalization or within 3 months of discharge. One of our assumptions (and the limitations of our pharmacy data) was that even a single antiprotease augmentation therapy prescription post-LT indicated indefinite post-LT therapy use.

To assess for selection bias, we compared the characteristics of LT recipients with AAT who had and did not have prescription fill information available through our pharmacy database.

### Transplant and perioperative characteristics

We compared recipient, donor, and transplant characteristics by recipient group (no augmentation, augmentation continuation, augmentation discontinuation, or augmentation commencement post-LT) using Fisher’s exact test for categorical variables and Wilcoxon rank-sum tests for continuous variables. Characteristics evaluated included recipient age, sex, Body Mass Index (BMI), blood type, lung allocation score, ischemia time, post-LT ventilator requirement, and postoperative stay, as well as donor age, sex, smoking status (>20 pack years), and cause of death. These transplant and perioperative characteristics are before the use (if any) of post-LT augmentation therapy.

### Post-LT long-term outcomes

Our primary outcomes were mortality and all-cause graft failure (ACGF; composite of mortality, graft failure, and retransplantation). Mortality and ACGF are reported to SRTR by individual transplant centers and this information is independently verified through linkage to the Social Security Master Death File (mortality) and waiting list (graft failure). We note that both the mortality and ACGF values may be subject to administrative censoring.

To analyze recipient characteristics, we compared the categorical variables (blood type, post-LT ventilator requirement) using Fisher’s exact test and continuous variables using a Wilcoxon rank-sum test.

To study the effect of post-LT use of augmentation therapy on mortality, we first created 2 populations of recipients. The first population (hereafter referred to as post-LT users) consisted of 32 recipients who continued AAT augmentation therapy post-LT as well as 13 recipients who did not use augmentation therapy pre-LT but started using it post-LT. The second population (hereafter referred to as post-LT nonusers) consisted of 77 recipients who used augmentation therapy pre-LT but discontinued it post-LT.

Ischemia was evaluated as a potential confounder but was not significantly correlated with survival time on Pearson correlation analysis and was not included in the final survivorship analysis. Kaplan-Meier curves for mortality and ACGF were plotted, including 95% confidence intervals, and statistical comparison was performed using the log-rank test. All recipients who reached the cutoff point of the study without a mortality or graft failure event were right-censored. To account for survivor bias, individuals not receiving post-LT therapy entered the at-risk group at 3 months post-LT and those who received post-LT therapy entered the at-risk group at 3 months post-LT or the time of the first prescription fill, whichever was greater.

Age was noted to be significantly correlated with death and ACGF. Therefore, we used restriction and matching techniques to ensure post-LT users and nonuser populations had similar mean ages (confirmed by Mann-Whitney U test) to limit confounding.

We then compared the mortality and ACGF outcomes between these restricted groups. Further, we used matching to estimate the average treatment effect (ATE). The ATE describes the expected effect of an intervention in an observational study or a randomized trial. The ATE describes what would be observed if everyone received a treatment and not just a subset of recipients. For observational studies, adjusting for potential confounders is key, as we have performed here with age. We binned the ages in groups of 40 to 49, 50 to 59, 60 to 69 and computed the average time to mortality in each binned group for the drug-continuing and drug-discontinuing populations. These averages were then averaged and their difference was taken.

### High-bilirubin subgroup analysis

As discussed in the Background, A1AT deficiency is primarily a liver disease with manifestations in the lung and liver. We hypothesized that there could be a benefit to post-LT use of AAT augmentation therapy in recipients with high disease burden pre-LT because LT does not address the root cause of AAT deficiency. To identify recipients with high pre-LT disease burden, we looked at the liver disease burden; the lung disease burden in all these recipients was high at the time of transplant (simply since they had end-stage lung disease necessitating transplant). We attempted to use several liver function parameters, such as aspartate aminotransferase, alanine aminotransferase, alkaline phosphatase, albumin, and bilirubin, but high levels of missingness necessitated us relying on bilirubin as a proxy for extent of liver disease. Bilirubin can be elevated in recipients with AAT deficiency due to endoplasmic reticulum stress and apoptosis of hepatocytes resulting in mild to severe hepatitis, cirrhosis, and fibrosis. However, liver manifestations of AAT deficiency need not be cholestatic.

We took the recipients in the 75th to 100th percentile of pre-LT bilirubin in the groups that used and did not use post-LT augmentation therapy. We also noted that these 2 groups had a similar distribution of pre-LT bilirubin. Kaplan-Meier analyses comparing mortality and ACGF in these 2 groups of recipients were performed with the same methods as described above. We then repeated these analyses by looking at recipients in the 50th to 100th percentile of pre-LT bilirubin who used and did not use post-LT augmentation therapy.

All analyses were performed using Python version 3.7.6 (Python Software Foundation, Fredericksburg, VA).

## Results

### Study population

From January 2011 to December 2021, we identified 23,433 LT recipients, 612 of whom had a diagnosis of AAT deficiency. Among the recipients with AAT deficiency, we obtained prescription fill information for 475 recipients (77.6% capture rate). Recipients who had prescription fill information available were younger (median [Q1-Q3]: 57 [52-63] vs 58 [52-63], *p* = 0.04), were less likely to acquire a post-LT infection (15.2% vs 27.7%, *p* = 0.001) without a significant increase in death from post-LT infection (5.3% vs 8.0%, *p* = 0.07) and their donors were less likely to have a >20 pack-year smoking history (6.7% vs 13.1%, *p* = 0.02) than those who did not have prescription fill information available. All other characteristics, such as recipient sex, BMI, waitlist time, and other donor characteristics, such as donor age and cause of death, were not significantly different between the 2 groups (Table S1).

**Figure 1:**
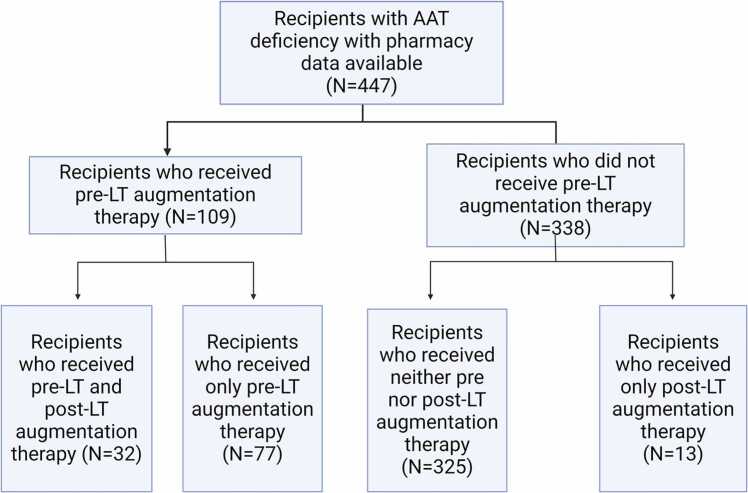
Patient cohort description and classification. AAT, alpha-1 antitrypsin; LT, lung transplantation.

After restricting to recipients with at least 3 months of post-LT follow-up available, we identified a final study population of 447 recipients. Among these recipients, 109 (24.4%) received AAT augmentation therapy pretransplant. Additionally, 13 recipients (2.9%) received augmentation therapy only post-LT. Figure 1 shows the recipient cohort and subpopulations considered.

### Perioperative outcomes by pre-LT use of AAT augmentation therapy

Compared to recipients who did not receive pre-LT augmentation therapy, recipients who received pre-LT augmentation therapy had longer post-LT hospital stays than those who did not (median [Q1-Q3]: 20 [13-28] vs 17 [12-24], *p* = 0.03) were more likely to receive a transplant from an older donor (median [Q1-Q3]: 33 [24-47] vs 29 [22-42], *p* = 0.03) and more likely to have an acute rejection episode (10.1% vs 2.5%, *p* = 0.001) without any increase in mortality from acute rejection (0% vs 1.2%, *p* = 0.58). Recipients who received pre-LT augmentation therapy were not significantly more likely to get a post-LT infection (11.0% vs 15.4%, *p* = 0.34) but were less likely to die from post-LT infection (0.9% vs 5.8%, *p* = 0.03). There were no significant differences in other factors, such as recipient age, sex, and waitlist time (Table 1).

**Table 1:** Characteristics of Transplant Recipients With Alpha-1 Antitrypsin Deficiency Categorized by Pretransplant Use of Alpha-1 Antitrypsin Augmentation Therapy

	Pretransplant augmentation therapy		*p*-value
Characteristic	Yes	No	
*Recipient characteristics*			
N	109	325^a^	
Age (years), median (Q1-Q3)	57 (53-63)	57 (52-62)	0.61
Female sex	48.4%	46.5%	0.83
BMI (kg/m^2^, mean)	24.8	24.6	0.66
Blood type			0.97
O	61 (50%)	147 (45.2%)	
A	48 (39.3%)	137 (42.2%)	
B	11 (9%)	31 (9.5%)	
AB	2 (1.7%)	10 (3.1%)	
Lung allocation score, median (Q1-Q3)	33.6 (33.0-35.1)	33.7 (33.0-34.7)	0.93
FEV1% predicted, median (Q1-Q3)	20 (17-26)	20 (16-26)	0.48
pCO_2_ in mm Hg, median (Q1-Q3)	47.55 (40-54)	47 (41-56)	0.42
Time on waitlist (years), median (Q1-Q3)	0.28 (0.09-1.02)	0.38 (0.09-1.02)	0.79
Unilateral transplant	13.9%	12.0%	0.62
Ischemia time (minutes), median (Q1-Q3)	320 (258-374)	333 (276-403)	0.07
Post-transplant ventilator requirement			0.91
None	2 (1.8%)	6 (1.8%)	
≤48 hours	82 (75.2%)	253 (77.8%)	
>48 hours, <5 days	15 (13.8%)	35 (10.8%)	
>5 days	10 (9.2%)	29 (8.9%)	
Unknown	0 (0%)	2 (0.7%)	
Length of postoperative stay in days, median (Q1-Q3)	20 (13-28)	17 (12-24)	**0.03**
Incidence of acute rejection	11 (10.1%)	12 (3.7%)	**0.02**
Death due to acute rejection	0 (0%)	4 (1.2%)	0.58
Incidence of post-LT infection	12 (11.0%)	50 (15.4%)	0.34
Death due to post-LT infection	1 (0.9%)	19 (5.8%)	**0.03**
*Donor characteristics*			
Age (years), median (Q1-Q3)	33 (24-47)	29 (22-42)	**0.03**
Female sex	22.2%	26.6%	0.36
Smoker	7.6%	5.6%	0.66
Donor cause of death			0.11
Anoxia	34 (31.2%)	97 (29.8%)	
Cerebrovascular/stroke	31 (28.5%)	60 (18.5%)	
Head trauma	42 (38.5%)	159 (48.9%)	
Other	2 (1.8%)	9 (2.8%)	

Abbreviations: AAT, alpha-1 antitrypsin; FEV1, forced expiratory volume in one second; LT, lung transplantation.

Characteristics that differed significantly at a threshold of *p < 0.05 are bolded. The table includes baseline as well as transplant and perioperative characteristics.*

a Excludes the 13 patients who started AAT augmentation therapy post-LT.

### Post-LT outcomes by post-LT use of AAT augmentation therapy

Of the 109 LT recipients who used prior prescription for AAT augmentation therapy, 32 (29.4%) continued augmentation therapy post-LT and 77 (70.6%) did not. Post-LT users were more likely to be younger (56.5 [53-59.75] vs 57 [53.75-63], *p* = 0.04) and had shorter ischemia times during transplant (mean 311 vs 363, *p* = 0.028) than post-LT nonusers. These differences are characterized in Table 2.

**Table 2:** Characteristics of Transplant Recipients With Alpha-1 Antitrypsin Deficiency by Use of Alpha-1 Antitrypsin Augmentation Therapy Post-Transplant

	Post-transplant augmentation therapy		*p*-value
Characteristic	Yes	No	
*Recipient characteristics*			
N	45	77	
Age (years), median (Q1-Q3)	56.5 (53-59.75)	57 (53.75-63)	**0.04**
Female sex	44.4%	50.6%	0.58
BMI (kg/m^2^, mean)	25.2	24.8	0.71
Blood type			0.08
O	28 (62.2%)	33 (42.9%)	
A	12 (26.7%)	36 (46.8%)	
B	5 (11.1%)	6 (7.8%)	
AB	0	2 (2.5%)	
Lung allocation score, median (Q1-Q3)	33.8 (32.9-34.6)	33.6 (33.0-34.9)	0.76
Time on waitlist (years), median (Q1-Q3)	0.22 (0.09-0.57)	0.21 (0.07-0.46)	0.19
Unilateral transplant	17.8%	11.7%	0.42
Ischemia time (minutes), median (Q1-Q3)	305 (245-363)	318 (273-375)	**0.03**
Post-transplant ventilator requirement			0.76
None	1 (2.2%)	1 (1.3%)	
≤48 hours	32 (71.1%)	59 (76.6%)	
>48 hours, <5 days	7 (15.6%)	10 (13%)	
>5 days	4 (8.9%)	7 (9.1%)	
Unknown	1 (2.2%)	0	
Length of postoperative stay in days, median (Q1-Q3)	22 (15-28)	20 (13-27)	0.33
Incidence of acute rejection	4 (8.9%)	8 (10.4%)	0.99
Death due to acute rejection	0 (0%)	0 (0%)	1.00
Incidence of post-LT infection	7 (15.6%)	9 (11.7%)	0.58
Death due to post-LT infection	1 (2.2%)	1 (1.3%)	1.00
*Donor characteristics*			
Age (years), median (Q1-Q3)	29 (24-44)	34 (25-47)	0.49
Female sex	22.2%	27.3%	0.67
Smoker	6.8%	3.9%	0.67
Donor cause of death			0.18
Anoxia	17 (37.8%)	20 (26%)	
Cerebrovascular/stroke	8 (17.8%)	26 (33.8%)	
Head trauma	19 (42.2%)	30 (39%)	
Other	1 (2.2%)	1 (1.2%)	

Abbreviation: LT, lung transplantation.

Characteristics that differed significantly at level *p < 0.05 are bolded.*^a^ The table includes baseline as well as transplant and perioperative characteristics.

a For this analysis, recipients with perioperative mortality were excluded and thus the total is 45 + 77 = 122 recipients (9 recipients with perioperative mortality excluded from the original 131).

Given the baseline differences in age and ischemia time between these 2 groups, we further investigated their relationship with mortality and ACGF. Age (Pearson *r* = −0.24, *p* = 0.01) but not ischemic time (Pearson *r* = −0.058, *p* = 0.56) was associated with post-LT mortality. Similarly, age (Pearson *r* = −0.22, *p* = 0.02) but not ischemic time (Pearson *r* = −0.053, *p* = 0.60) was associated with ACGF.

**Figure 2:**
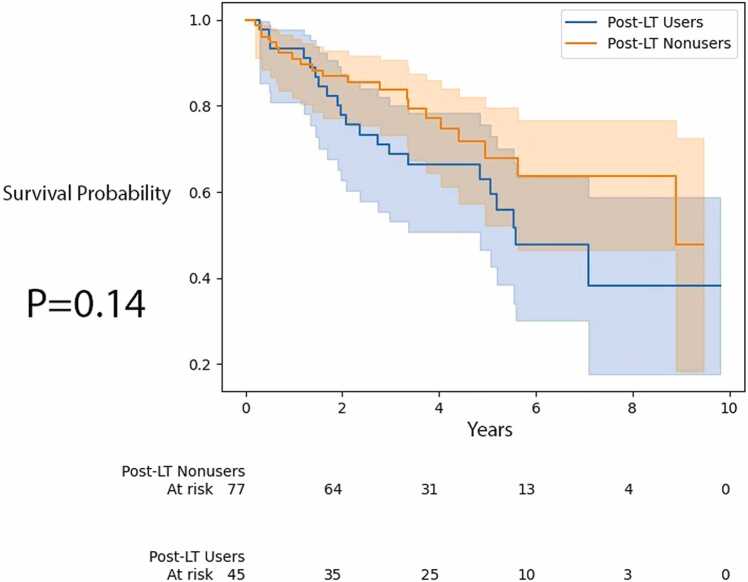
Time to mortality for recipients by post-LT AAT augmentation therapy use. AAT, alpha-1 antitrypsin; LT, lung transplantation.

**Figure 3:**
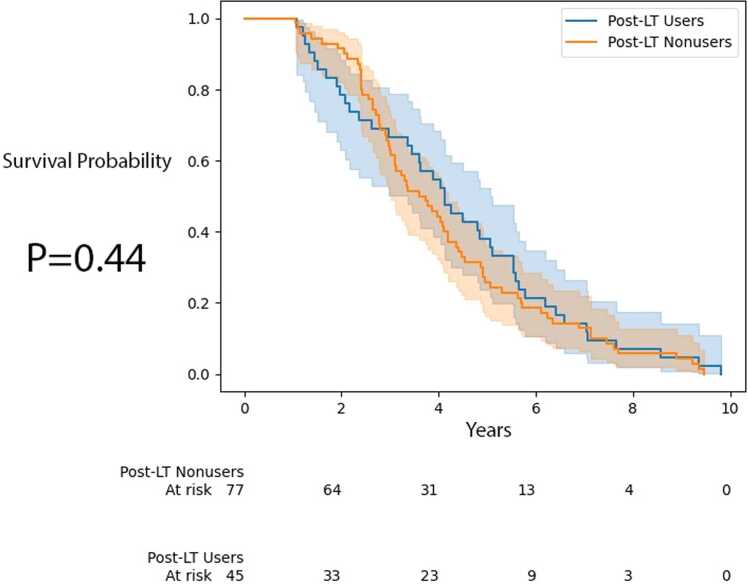
Time to ACGF for recipients by post-LT AAT augmentation therapy use. AAT, alpha-1 antitrypsin; ACGF, all-cause graft failure; LT, lung transplantation.

Further, we performed Kaplan-Meier survival curve analysis ( Figures 2 and 3) to look at post-LT time to mortality and ACGF for post-LT users and nonusers. There was no significant difference in time to mortality and ACGF (*p* = 0.13 and *p* = 0.44 respectively, by log-rank test).

We then performed age-adjusted estimation of the ATE and calculated +1.69 years on time to mortality and +1.48 years on time to ACGF.

### High-bilirubin subgroup analyses

**Figure 4:**
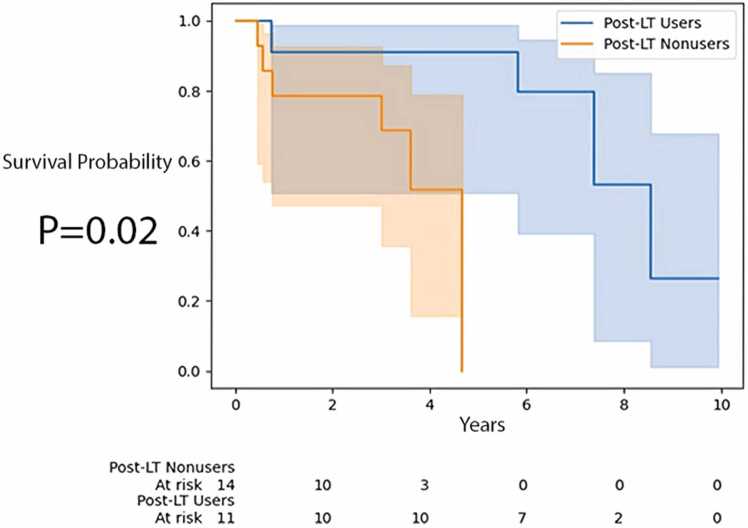
Time to mortality for recipients in the 75th to 100th percentile of serum bilirubin by post-LT AAT augmentation therapy use. AAT, alpha-1 antitrypsin; LT, lung transplantation.

**Figure 5:**
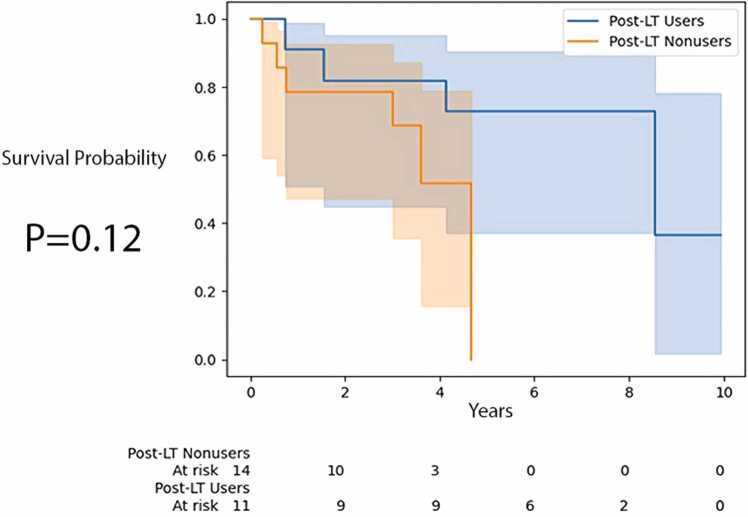
Time to ACGF for recipients in the 75th to 100th percentile of serum bilirubin by post-LT AAT augmentation therapy use. AAT, alpha-1 antitrypsin; ACGF, all-cause graft failure; LT, lung transplantation.

The Kaplan-Meier survival curve analysis of recipients with serum bilirubin in the 75th to 100th percentile is shown in Figures 4 and 5. Post-LT users with serum bilirubin in the 75th to 100th percentile had longer time to mortality than post-LT nonusers (*p* = 0.02, log-rank test). However, time to ACGF was not statistically different between these 2 groups (*p* = 0.12, log-rank test). A comparison of recipients with serum bilirubin in the 50th to 100th percentile who used augmentation therapy post-LT vs those who did not use it was not significant (not shown), with *p* = 0.34 for time to mortality and *p* = 0.31 for time to ACGF.

## Discussion

In this national study of AAT augmentation therapy for lung transplant recipients with AAT deficiency, we found that only 109 of 447 recipients with AAT deficiency had evidence of outpatient AAT augmentation therapy pre-LT, and that younger recipients were more likely to have received augmentation therapy. While on unadjusted analysis, we did not observe a difference in survival by post-transplant use of augmentation therapy, after matching for age, use of augmentation therapy post-transplant was associated with an additional 1.69 years of survival. Finally, recipients with pre-LT bilirubin in the top quartile benefitted from use of AAT augmentation therapy post-LT.

The current guidelines recommend augmentation therapy for patients with AAT deficiency (homozygous genotype) with forced expiratory volume in one second (FEV1) <65%.^9^ All the LT recipients in this study had pre-LT FEV1 of far less than 65%. We note that there was no significant difference between pCO_2_ and FEV1% predicted in recipients who received and did not receive AAT augmentation therapy. This suggests there may be a need for greater standardization of care for patients with AAT deficiency who develop advanced lung disease or could indicate potential disparities in patient access to therapy, both of which warrant further investigation. To ensure that everyone who should be getting augmentation is receiving it, we support standardization of care for these patients with genetic testing to follow-up low serum AAT levels to determine eligibility for therapy.^9^ If started expeditiously, the therapy has the potential to delay the emphysematous changes that lead to end-stage lung failure. Future studies could use registries more specific to AAT and look at genotype and access to medical therapy. We would also like to highlight work by organizations, such as the Alpha-1 Foundation, in expanding access to medical care for patients with AAT deficiency and attempting to reduce socioeconomic disparities that affect access to care for these patients.^11^ We acknowledge, however, that coding of the diagnosis of AAT deficiency may be clouded by potential heterozygote genotypes being included as well (limitations) and that the current guidelines do not recommend augmentation therapy for heterozygous genotypes with emphysema.^9^

We estimated that continuation of augmentation therapy post-LT was associated with 1.69 years longer survival after transplant, even after adjusting for recipient age. This suggests that there may be a benefit to continuation of augmentation therapy post-LT. Future research will benefit from confirmation in other populations through prospective studies of post-LT recipients with AAT deficiency.^2^, ^9^, ^12^, ^13^

Given our hypothesis that recipients with higher AAT deficiency disease burden pre-LT might benefit from use of augmentation therapy post-LT, we performed subgroup analysis on recipients by pre-LT serum bilirubin in the top quartile (as a proxy for disease burden). We found that recipients with AAT with serum bilirubin in the top quartile could have potential survival benefit from use of augmentation therapy post-LT. However, the comparison of recipients with pre-LT serum bilirubin in the 50th to 100th percentile who used vs did not use post-LT augmentation therapy was not significant, suggesting that only recipients with higher disease burdens may benefit from augmentation therapy post-LT. To our knowledge, there have been no studies or guidelines regarding the use of augmentation therapy in this subpopulation. Further studies with larger populations could define specific bilirubin cutoffs for more personalized treatment.

This study has several limitations. First, the primary limitation of this study is its design; it is a registry-based retrospective study. The registry reflects several institutions across the United States and heterogeneous surgical and medical protocols and follow-up procedures. The augmentation therapy data were drawn from a separate database which lacked information on frequency, dosage, and other pharmacological parameters. The retrospective nature of the analysis and the limited sample size may involve potential confounding or potential selection bias. Since we used a 3-month window to ascertain whether recipients used augmentation therapy post-LT (our primary exposure), we could not assess outcomes (such as acute rejection) occurring in the 3-month period. While our pharmacy database is one of the largest prescription databases in the United States, it only contains outpatient prescriptions and does not include potential treatment options other than AAT augmentation, such as gene therapy or medications in clinical trials. Additionally, these data were only available to us through 2021, and we were unable to assess whether medication discontinuation was due to changes in insurance that might result in differential capture of prescription fills. However, such misclassification would likely bias the results toward the null, making ours a conservative estimate of the benefit associated with augmentation therapy. Second, this is a small subset due to the rarity of this disease. Despite drawing from 10 years of transplant recipients, we were able to identify only 612 recipients with a diagnosis of AAT, of whom 475 had prescription data available. The sample size limits our ability to adjust for confounders with more sophisticated analyses. In addition, as AAT deficiency only has 1 International Classification of Diseases code (E88.01), it may potentially include carriers of the Z allele (allele associated with very low AAT levels in the blood; homozygous ZZ patients are at high risk of developing emphysema and liver disease) who are then documented as receiving a transplant due to a diagnosis of AAT deficiency. As centers begin performing increasing numbers of transplants, the increased availability of data will allow larger retrospective studies, prospective studies, and randomized trials. Third, AAT deficiency liver manifestations may not always be cholestatic and thus bilirubin may not be the most reliable indicator of liver disease. However, other liver tests, such as those used to calculate a model for end-stage liver disease score (international normalized ratio, creatinine, and Na), were not consistently available for the recipients. Still, we believe that our findings are an important first step toward understanding the role of augmentation therapy in post-LT care for recipients with AAT deficiency.

In conclusion, we report on the largest retrospective study of pre- and post-LT augmentation therapy use in recipients with AAT deficiency. Use of augmentation therapy was not associated with available recipient characteristics, indicating its use may currently reflect differences in provider philosophy or access to treatment. We found that continuing augmentation therapy post-LT was associated with longer survival, especially among recipients with elevated pre-LT bilirubin. These findings should help inform discussions between providers and LT candidates and recipients about the decision to use or continue augmentation therapy. We hope that future prospective, phase 3 studies will address this topic in further detail and prompt discussions regarding national guidelines.

## Ethical considerations

The study was deemed exempt from the need for Institutional Review Board approval by the Johns Hopkins Institutional Review Board NA_00042871.

## Author contribution

Conceptualization: Oak, Ruck, Bush. Data acquisition: Oak, Ruck, Massie, Segev. Drafting of the manuscript: Oak, Ruck. Critically revising the manuscript: all authors. Statistical analysis: Oak, Ruck, Massie. Study supervision: Bush.

## Disclosure statement

Allan Massie and Dorry Segev report financial support was provided by the National Institutes of Health. The other authors declare that they have no known competing financial interests or personal relationships that could have appeared to influence the work reported in this paper.

This study was funded by the 10.13039/100000049National Institute on Aging (NIA) F32 grant F32AG067642 (Ruck), R01DK132395, (Massie) and K24AI144954 (Segev). The data reported here have been supplied by the Hennepin Healthcare Research Institute (HHRI) as the contractor for the Scientific Registry of Transplant Recipients (SRTR).

The views expressed in this manuscript are solely of the authors and do not reflect the views of the United States Air Force or the Department of Defense. The interpretation and reporting of these data are the responsibility of the author(s) and in no way should be seen as an official policy of or interpretation by the SRTR or the U.S. Government.

This work was accepted as a full-length oral presentation at ISHLT 2024.

## Data Availability

The data that support the findings of this study can be made available upon reasonable request.

## Footnotes

Appendix A Supplementary data associated with this article can be found in the online version at doi:10.1016/j.jhlto.2024.100201.
