# Supplementary material for: Lung transplant outcomes for recipients with alpha-1 antitrypsin deficiency, by use of alpha-1 antitrypsin augmentation therapy
Source: JHLT Open. 2024 Dec 24;7:100201. doi: 10.1016/j.jhlto.2024.100201 (PMC11935422; doi:10.1016/j.jhlto.2024.100201)
Supplement: Supplementary file 1 — Supplementary material [file mmc1.docx]

Supplementary Table 1: **Characteristics of transplant recipients with Alpha-1 Antitrypsin Deficiency by availability of prescription fills.** Characteristics that differed significantly at level p<0.05 are bolded. The table includes baseline as well as transplant and perioperative characteristics.

|  | **Prescription data available in Symphony database** | | **p-value** |
| --- | --- | --- | --- |
| **Characteristic** | **Yes** | **No** |  |
| **Recipient Characteristics** |  |  |  |
| N | 475 | 137 |  |
| Age (years), median (Q1-Q3) | 57 (52-63) | 58 (53-63) | **0.04** |
| Female sex | 46.5% | 38.0% | 0.08 |
| BMI (kg/m^2^, mean) | 24.8 | 24.4 | 0.31 |
| Blood Type |  |  | 0.97 |
| O | 221 (46.5%) | 61 (44.5%) |  |
| A | 196 (41.3%) | 60 (43.8%) |  |
| B | 45 (9.5%) | 13 (9.5%) |  |
| AB | 13 (2.7%) | 3 (2.2%) |  |
| Lung allocation score (mean) | 33.6 (32.9-35.0) | 33.7 (32.8-35.0) | 0.78 |
| Time on waitlist (years), median (Q1-Q3) | 0.34 (0.09-1.06) | 0.33 (0.07-1.09) | 0.83 |
| Unilateral transplant | 12.2% | 13.9% | 0.66 |
| Ischemia time (minutes), mean (Q1-Q3) | 323 (259-378) | 309 (244-362) | 0.18 |
| Post-transplant ventilator requirement |  |  | 0.26 |
| None | 9 (1.9%) | 3 (2.2%) |  |
| <=48 hours | 356 (74.9%) | 99 (72.3%) |  |
| >48 hours, <5 days | 56 (11.8%) | 14 (10.2%) |  |
| >5 days | 51 (10.7%) | 17 (12.4%) |  |
| Unknown | 3 (0.7%) | 4 (2.9%) |  |
| Length of postoperative stay (days), median (Q1-Q3) | 22 (15-28) | 20 (13-27) | 0.99 |
| Incidence of Acute Rejection | 24 (5.1%) | 11 (8.0%) | 0.21 |
| Death due to Acute Rejection | 3 (0.6%) | 2 (1.4%) | 0.31 |
| Incidence of post-LT infection | 72 (15.2%) | 38 (27.7%) | **0.001** |
| Death due to post-LT infection | 25 (5.3%) | 11 (8.0%) | 0.07 |
| **Donor Characteristics** |  |  |  |
| Age (years), median (Q1-Q3) | 30 (23-43.5) | 32 (23-48) | 0.27 |
| Female sex | 22.3% | 19.7% | 0.56 |
| Smoker | 6.7% | 13.1% | **0.02** |
| Donor cause of death |  |  | 0.23 |
| Anoxia | 142 (29.9%) | 32 (23.4%) |  |
| Cerebrovascular/Stroke | 97 (20.5%) | 38 (27.7%) |  |
| Head Trauma | 223 (46.9%) | 63 (46%) |  |
| Other | 13 (2.7%) | 4 (2.9%) |  |
